# CLOUD: a non-parametric detection test for microbiome outliers

**DOI:** 10.1186/s40168-018-0514-4

**Published:** 2018-08-06

**Authors:** Emmanuel Montassier, Gabriel A. Al-Ghalith, Benjamin Hillmann, Kimberly Viskocil, Amanda J. Kabage, Christopher E. McKinlay, Michael J. Sadowsky, Alexander Khoruts, Dan Knights

**Affiliations:** 1 MiHAR lab, Université de Nantes, 44000 Nantes, France; 2 Department of Computer Science and Engineering, University of Minnesota, Minneapolis, MN 55455 USA; 3 Division of Gastroenterology, Hepatology, and Nutrition in the Department of Medicine, University of Minnesota, Minneapolis, MN USA; 4 Biotechnology Institute, University of Minnesota, St. Paul, MN 55108 USA; 5 Department of Soil, Water, and Climate, University of Minnesota, St. Paul, MN 55108 USA; 6 Department of Emergency Medicine, CHU Nantes, Nantes, France

**Keywords:** Outlier, Fecal microbiota transplantation, Microbiome, Conformity, Stability, Dysbiosis

## Abstract

**Background:**

Dysbiosis of the human gut microbiome is defined as a maladaptive or clinically relevant deviation of the community profile from the healthy or normal state. Dysbiosis has been implicated in an extensive set of metabolic, auto-immune, and infectious diseases, and yet there is substantial inter-individual variation in microbiome composition even within body sites of healthy humans. An individual’s microbiome varies over time in a high-dimensional space to form their personal microbiome cloud. This cloud may or may not be similar to that of other people, both in terms of the average microbiome profile (conformity) and the diameter of the cloud (stability). However, there is currently no robust non-parametric test that determines whether a patient’s microbiome cloud is an outlier with respect to a reference group of healthy individuals with widely varying microbiome profiles.

**Methods:**

Here, we propose a test for outliers’ detection in the human gut microbiome that accounts for the wide range of microbiome phenotypes observed in a typical set of healthy individuals and for intra-individual temporal variation. Our robust nonparametric outlier detection test, the CLOUD test, performs two assessments of a patient’s microbiome health: conformity, the extent to which the patient’s microbiome cloud is ecologically similar to a subset of healthy subjects; and stability, which compares the cloud diameter of a patient to those of healthy subjects. The CLOUD test is based on locally linear embedded ecological distances, allowing it to account for widely varying microbiome compositions among reference individuals. It also leverages temporal variability within patients and reference individuals to increase the robustness of the test.

**Results:**

We describe the CLOUD test, and we apply it to one novel and two previously published cohorts of patients receiving fecal microbiota transplantation for recurrent *Clostridium difficile* colitis, as well as to two known healthy cohorts, demonstrating high concordance of the CLOUD conformity and stability indices with clinical outcomes.

**Conclusions:**

Although the CLOUD test is not, on its own, a test for clinical dysbiosis, it nonetheless provides a framework for outlier testing that could be incorporated into evaluation of suspected dysbiosis, which may play a role in diagnosis and prognosis of numerous pediatric and adult diseases.

**Electronic supplementary material:**

The online version of this article (10.1186/s40168-018-0514-4) contains supplementary material, which is available to authorized users.

## Background

The human gut microbiome is known to be highly variable between individuals, as well as within individuals over time [1, 2]. Substantial methods development has resulted in better discriminative tests for the microbiome, in which the goal is to identify specific taxa that differentiate treatment groups or correlate with experimental variables or clinical metadata [3–7]. These supervised tests are useful when a study has two or more experimental groups, or a known biochemical gradient related to the microbiome.

Halfvarson et al. recently defined a two-dimensional healthy plane, calculated in a space derived from principal coordinates analysis (PCoA) of unweighted UniFrac distances of healthy subjects, using the least-squares method. This plane was then used as a proxy to represent the normal microbial variation within healthy subjects and to summarize the abnormal, intermittent dysbiosis associated with inflammatory bowel disease (IBD). The authors found that microbiomes of IBD patients fluctuated more than those of healthy individuals and, at times, occupied a different region of PCoA space, based on deviation from the newly defined healthy plane [8]. This approach represents a significant advance in dysbiosis testing and is likely to be effective in cases with relatively homogeneous and unimodal reference populations.

There may be different situations where a model is desired that can account for widely varying reference populations. Indeed, the human microbiome is highly multivariate, and health can be associated with many different taxonomic configurations that may not be captured by a plane or hyperplane. There are currently no known non-parametric tests for microbiome outliers, defined as significant deviation of the community profile, in ecological distance space, from those of a large reference group of healthy subjects. Such a test will be important in medical microbiome research for comparing a patient’s microbiome to a reference population to determine when it is significantly abnormal or dysbiotic in terms of conformity or stability, without a priori knowledge of the dysbiotic state.

Here, we present the Cloud-based LOcally linear Unbiased Dysbiosis (CLOUD) test, a generalized robust non-parametric test for dysbiosis that utilizes the full high-dimensional between-sample ecological distance matrix. Ultimately, this test could be incorporated into clinical practice to enhance microbiome-based diagnostics and decision-making.

## Methods

### Description of the CLOUD test

**Fig. 1:**
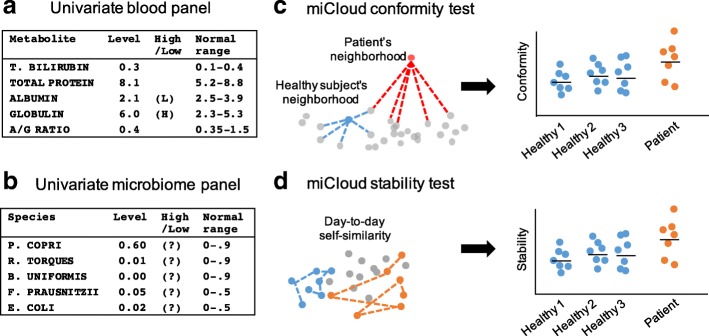
Comparison of univariate blood and microbiome tests with the multivariate CLOUD test. **a**, **b** In contrast to a univariate blood panel, in which healthy ranges for individual metabolites are well defined, the normal ranges for individual bacterial species in the human microbiome are too wide to be meaningful, with many taxa being completely absent from some individuals’ guts and dominating other individuals’ guts. **c**, **d** In contrast, CLOUD uses a high-dimensional representation of the whole microbiome profile to define the normal range of healthy microbiomes

One major challenge in developing a generalized test for dysbiosis is that human gut microbial composition is highly different across individuals, with some healthy individuals having almost completely different sets of taxa than others [9–13]. Thus, measures of ecological similarity at the whole-community level are a reasonable alternative to conventional univariate tests such as those used in blood. A typical blood test reports levels of individual blood metabolites and classifies them as normal or abnormal according to the normal range in a healthy individual (Fig. 1a). This type of univariate test works when each variable or metabolite has a relatively well-defined normal range. However, the individual species in the human gut microbiome can vary widely in relative abundance from individual to individual, making it impossible to define a healthy normal range (Fig. 1b).

Thus, our objective is to build an unsupervised multidimensional test that will allow the classification of a complete microbiome profile either as sufficiently healthy or as an outlier, in comparison to a reference cohort of healthy subjects. This test takes into account the following three challenges:

i) The human microbiome is highly multivariate
ii) The healthy human gut microbiome has many different taxonomic configurations
iii) An individual’s microbiome can vary substantially from day to day

**Fig. 2:**
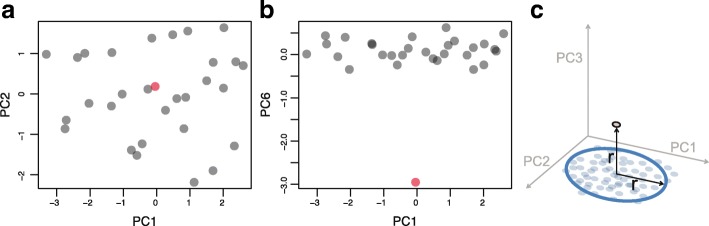
**a** First two dimensions of principal coordinates analysis (PCoA) of a simulated outlier and 29 reference microbiomes. Even though the outlier has no species in common with the reference samples, two-dimensional PCoA obscures the fact that it is an outlier, **b** PC6 PCoA axe plotted against PC1, showing that in this particular case six dimensions are sufficient to observe the outlier. In real clinical data, the true number of dimensions required is not known. **c** Example of an outlier that would not be detected by a centroid-based test

We propose the non-parametric CLOUD test to address these issues. Specifically, to address point (i), the CLOUD test uses multivariate ecological measures of whole-community dissimilarity in place of univariate tests of individual species (Fig. 1c). Comparisons of microbiomes must necessarily be highly dimensional, because low-dimensional embeddings of outliers in a reference distribution, such as with PCoA, can completely obscure an outlier, even when there are no shared taxa between the outlier and the reference samples, as shown in Fig. 2a, b.

It is important to measure both the conformity (similarity in profile to healthy individuals) and the stability (consistency of profile over time relative to the consistency of healthy individuals’ profiles over time) of a patient’s microbiome. Testing for conformity of an individual microbiome profile compared to reference microbiome profiles is a non-trivial problem. Microbiome distributions across healthy individuals can occupy arbitrary density distributions in high-dimensional microbiome space. These distributions may have curvature, gaps/clusters, multiple modes, and long gradients [10, 11]. Thus, typical parametric measures of conformity, such as multivariate normal distributions or the Mahalanobis distance, do not suffice to capture these complex, arbitrary, and high-dimensional density distributions [14–16]. On the other hand, simple centroid-based tests, in which test samples are compared to the centroid of the normal distribution cloud, can also obscure outliers depending on the shape of the reference cloud, as shown in Fig. 2c.

### A nonparametric test for microbiome outliers using local ecological distances

To address point (ii) above, the CLOUD test uses only local ecological distances (UniFrac distances or Bray-Curtis distances) to assess the similarity of a test point to the reference cloud rather than point-to-entire-distribution distances. The procedure is as follows:

1. For each reference subject *i = 1…n* in the reference population of size *n*, identify the *k* nearest neighbors also in the reference population. Calculate the *d*_*i*_, the diameter of the neighborhood, as the average ecological distance from that subject to the *k* neighbors. *k* is typically chosen as 5% of the total size of the reference set.
2. Calculate the average neighborhood diameter $\overline{d}=\frac{\sum_{i=1}^n{d}_i}{n}$.
3. For each reference subject *i = 1…n*, calculate the ratio of that subject’s neighborhood diameter to the average neighborhood diameter, ${r}_i=\frac{d_i}{\overline{d}}$ . This ratio is the outlier detection test.
4. Identify the *k* nearest neighbors of the test sample in the reference population. Calculate *d*_*j*_, the average ecological distance from the test subject to its *k* nearest reference neighbors, and the outlier detection test, the ratio of that subject’s neighborhood diameter to the average neighborhood diameter in the reference group: ${r}_j=\frac{d_j}{\overline{d}}$.
5. Calculate an empirical outlier percentile for the test subject as the fraction of reference outlier detection test greater than or equal to the test subject’s outlier detection test.

In other words, a person’s microbiome is considered normal if it is sufficiently close to at least a small number of other normal people and dysbiotic if it deviates from this relationship. The detailed R code used for calculating the neighborhood diameter is available in Additional file 1. An outlier percentile of 0.05, for example, indicates that the test subject is more distant from their nearest *k* reference neighbors than 95% of reference subjects are from their nearest *k* reference neighbors. The outlier detection test statistic *r* also has a simple and useful interpretation. A subject with outlier detection test *r* = 2 has a neighborhood diameter that is twice as large as the average neighborhood diameter in the reference population.

**Fig. 3:**
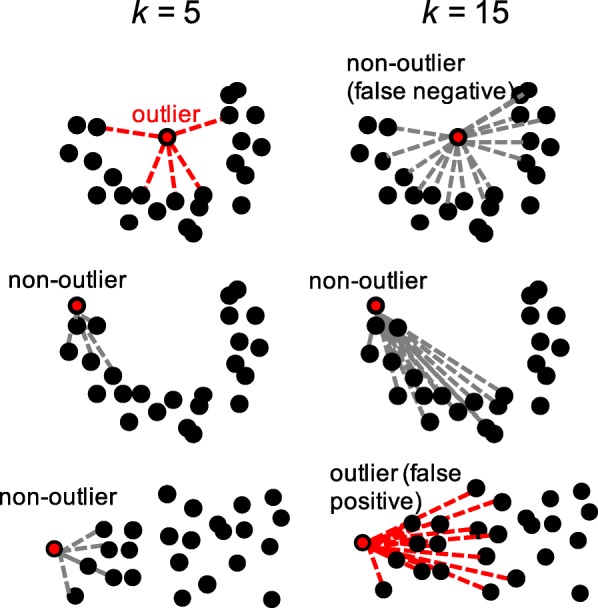
Graphical illustration of how with certain high-dimensional manifolds setting *k* too high can cause actual outliers to be classified as normal (false negative) and can cause normal points to be classified as outliers (false positive). Using large *k* approaching *n* defeats the purpose of the local distance measure, which is to allow the test to use only local regions in ecological distance space and can cause normal reference samples at the extremes of the distributions to be classified as outliers. On the other hand, if *k* is too small, then it is not robust to subtle variations in the reference group. By default, the CLOUD test sets the neighborhood size to 5% of the size of the reference set

An important feature of the CLOUD test is that it leverages only very local distances in the ecological distance space. This enables it to account, non-parametrically, for arbitrary density distributions in the highly dimensional landscape of healthy microbiomes (Fig. 3). Larger values of *k* are typically associated with increasing numbers of putative outliers, even within the reference distribution (Fig. 3). For this and other tests described below, setting *k* to be close to the size of the full data set allows the most conservative identification of outliers from a clinical perspective. However, values of *k* that are much smaller than the total number of subjects allow the test to account for larger global variation in normal microbiome profiles. Thus, *k* can be thought of as a smoothing parameter on the shape of the high-dimensional reference microbiome cloud. In general, *k* should be at least larger than the number of expected outliers in the reference distribution. In our standard test, we set *k* to 5% of the total number of reference samples when testing individual samples, and 5% of the total number of reference subjects when averaging distances across samples within each subject. We also tested several *k* values, corresponding to a range from 5 to 80% of the cohort, on several data sets described below, and found that the results are not especially sensitive to the choice of *k*.

The purpose of using *k* < *n* is a key component of the CLOUD test as it allows the flexibility of the test with respect to arbitrary shapes in the high-dimensional manifold on which the reference population’s ecological distances lie. Larger sample sizes are always important to increase power. The way that a larger sample size benefits the CLOUD test is at the level of *n*, the size of the reference population. The entire reference population is available to be used for the testing of any single test subject to determine whether it is an outlier, but the use of local neighborhoods is essential to avoid the pitfalls associated with centroid-based tests as shown graphically in Fig. 2.

To address point (iii) above, we also present here the CLOUD stability test (Fig. 1d). As with the conformity test, the stability test is performed separately on each test subject from a cohort of test subjects. We calculate day-to-day stability using self-similarity, by measuring the ecological distance (e.g., Unweighted UniFrac distance) of a subject on 1 day to that subject’s previous day. The average of all day-to-day UniFrac self-distances of a test subject is then compared to the distribution of average day-to-day UniFrac self-distances of the reference subjects to obtain an empirical outlier percentile in the same manner as the conformity test.

### Patients and donors

We analyzed several published data sets as well as novel samples from a reference population. These included five patients from a published dataset who suffered from multiply recurrent *Clostridium difficile* infection (CDI) refractory to standard antibiotic therapies (patients CD1 to CD5) and were treated with fecal microbiota transplantation (FMT) [9]. Among the five patients (CD1 to CD5) with recurrent CDI who received FMT, four were cured following FMT and one failed.

Sixteen healthy subjects who participated as standard stool donors in the University of Minnesota Microbiota Therapeutics Program also participated in this study. Inclusion and exclusion criteria for stool donor qualifications were described previously [10]. Briefly, in addition to qualifying as blood donors, these individuals took no medications; had no history of recent (< 6 months) antibiotics exposure; had no gastrointestinal, immunologic, neurodevelopmental, or psychiatric problems; had a body mass index of < 25 kg/m2; and had normal metabolic testing. The Institutional Review Board (IRB) at the University of Minnesota approved prospective collection of fecal specimens and their analysis.

### Fecal microbiota transplantation

FMT was performed using a standardized preparation of concentrated fresh or frozen fecal bacteria via colonoscopy as previously described [17, 18]. All patients were treated with oral vancomycin, 125 mg four times daily, until 2 days prior to the procedure. The day before the procedure, patients received a polyethylene glycol-based colonoscopy prep (GoLYTELY® or MoviPrep®) to remove residual antibiotics and fecal material. Donor fecal microbiome was placed into the terminal ileum and/or cecum via the biopsy channel of the colonoscope.

### Sample collection

Fecal samples were collected using swabs to obtain feces deposited into a toilet hat immediately after production. Samples were subsequently transferred to the laboratory, processed as previously described and stored at − 80 °C until used [18]. A total of 96 samples were collected from day − 2 (2 days pre-FMT) to day 151 (151 post-FMT) from the 4 patients who were cured of recurrent CDI by the FMT procedure. Moreover, 59 post-FMT samples from patient CD5, who failed to be cured by FMT, were collected. We also collected 247 fecal samples in healthy subjects longitudinally, from day 1 (first day of the collection) to day 75.

### DNA extraction, PCR, sequencing, and sequence processing and analysis

After fecal DNA isolation (MoBio, Carlsbad, CA fecal DNA kit), amplicons spanning the V4 region of bacterial 16S rRNA were generated and sequenced using an Illumina MiSeq platform at the University of Minnesota Genomic Center, Minneapolis, MN, (USA). Amplicons were sequenced in 2 × 250 paired-end mode. The 16S rRNA sequencing data from the Illumina runs were quality controlled, trimmed, and demultiplexed as implemented in Quantitative Insights Into Microbial Ecology (QIIME 1.8.1) [19] and the Illumina demultiplexing and processing protocol [20] with current quality-filtering recommendations [21]. After quality control and demultiplexing, we picked closed reference OTUs at a 97% similarity cut-off against Greengenes database version 13_8 [22]. Following trimming and quality filtering from a total of 49,521,442 sequences, we randomly subsampled to 5652 sequences/sample in order to normalize read depth across all samples. All further analyses were performed using this rarefied read depth. Sequences were then analyzed by using unweighted UniFrac, followed by PCoA [23]. Statistical analyses were performed with R version 3.4.0 (2017-04-21) [24].

## Results

### Interpretation of the CLOUD test

The CLOUD test provides an outlier percentile for the null hypothesis that a single predetermined test subject’s microbiome profile is drawn from an independent reference population. The outlier percentile describes the probability of a randomly chosen healthy subject having as large a neighborhood size as that of the test subject. The outlier percentile is determined by the empirical distribution of neighborhood sizes within the reference population. The repeated random sampling is the set of reference subjects included in the reference population. One may consider an analogy to assigning an outlier percentile to a person’s physical height based on the distribution of physical heights observed in a reference population of people. If the heights of the people in the reference population are normally distributed, then one may use a normal distribution to assign an outlier percentile to the test subject. This outlier percentile would describe what fraction of the reference subjects have a height greater than or equal to the height of the test subject, under the assumption that the reference subject heights followed a normal distribution with certain parameters. If this normality assumption were to be false for a particular reference population, and if the reference population were sufficiently large to obtain small outlier percentiles and were sufficiently unbiased to represent a truly random sampling of the total reference population, then one may instead use the empirical distribution of heights in the reference group to obtain and empirical outlier percentile for the independent test subject. In the same way, the CLOUD test outlier percentile is simply the fraction of reference subjects whose local neighborhood diameter is greater than or equal to the neighborhood diameter of the independent test subject.

Importantly, our test is not designed for identifying outliers from within the reference population, although we do perform hold-out cross-validation to assess outlier status in a healthy population consisting of people from three different countries as a demonstration of the flexibility of the CLOUD test with respect to clustering and high multivariate variation in the reference group. There is a history of established statistical tests that are designed to identify outliers within a given reference group. These include Grubbs’ Test [25] for testing whether there is a single outlier, the Tietjen-Moore test [26] for testing whether there is a specific number of outliers, and the Generalized extreme Studentized deviate test [27] for testing whether there is any number, below a certain upper bound, of outliers present in a group of otherwise normally distributed reference values. In contrast to these tests, the CLOUD test assumes that the reference set does not have outliers and is instead designed to test whether a single new independent subject is an outlier based on the reference set. In additional contrast to the aforementioned established tests, the CLOUD test is multivariate, non-parametric, making no assumptions about the distribution of reference values, and based on ecologically informed distance metrics specifically designed for comparing compositions of communities.

### Dimensionality of the ecological distance matrix with respect to taxonomy profiles

In contrast to outlier detection methods that use a small number of dimensions of principal coordinates analysis (PCoA) space, the CLOUD test does use a full-rank version of the ecological distance matrix, without any low-rank approximations. The distance metric itself is a transformation of the data from a P-dimensional space, where P is the number of taxa in the microbiome profile, to an “*N* − 1” dimensional space, where *N* is the number of samples. Depending on the size of the reference population, *P* may at times be substantially larger than *N*, and the distance transformation would represent an embedding of the taxonomy profiles into a lower dimension space. For example, if there were 1000 taxa in a data set with only 100 samples, then the ecological distance matrix may have lower rank than the taxon profile matrix; however, there are often many correlated groups of taxa in the taxon profile matrix, such that the actual rank of the taxon profile matrix may be less than the number of unique taxa observed. Thus, the CLOUD test that uses the full ecological distance matrix does not necessarily utilize the full dimensionality of the taxon profile space but does utilize a substantially higher number of dimensions than a test that operates in only a small number of PCoA dimensions.

### Application 1: conformity tests in healthy subjects

**Fig. 4:**
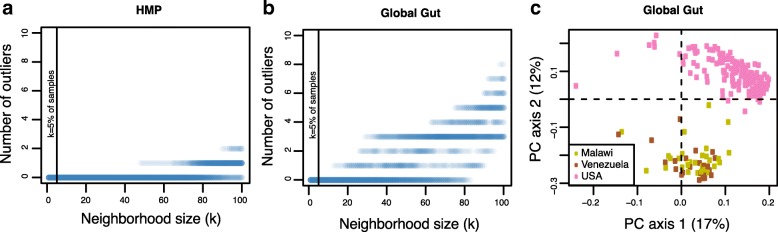
**a** Number of nearest healthy neighbors chosen in the CLOUD test prediction to find outliers in an international cohort (the HMP data set). We randomly selected a test dataset of 50 subjects and randomly selected a training dataset of 100 subjects 30 times in the full dataset of 200 subjects. We repeated the random selection of the training dataset 30 times. We did not identify outliers, excepting for extreme value of *k* in several random training datasets. This analysis demonstrates the robustness of the CLOUD test to the neighborhood size. The vertical bar represents 5% of the training dataset, the default neighborhood size for the test. **b** The same analysis was performed for the Global gut data set. **c** Principal coordinate plot of the Global gut dataset, from Unweighted UniFrac distance, demonstrating that the CLOUD test is robust to strong clustering effects with a reference group

To assess the ability of our test to identify healthy individuals given a widely varying reference population, we used two large-scale microbiome data sets to populate the multidimensional landscape with healthy microbiomes. We then used hold-out testing to evaluate the type I error rates of the test with repeated subsampling of these reference populations into separate “reference” and “test” groups. First, we analyzed 16S rRNA gene-based data (variable regions V3-V5) from the Human Microbiome Project (HMP), including 239 healthy subjects [10]. The data are available at https://www.hmpdacc.org/. In this dataset, we used a subset of the gut samples, excluding samples from obese patients, leaving 200 samples from 200 patients. Full metadata and annotation protocols are available on the HMP DACC website (https://www.hmpdacc.org/HMMCP/). We used the unweighted UniFrac distance matrix of the 200 fecal samples as the ecological distance matrix. Although the CLOUD test is designed for comparing a test subject to an independent reference group, we desired to assess the outlier status of subsets of the reference population with respect to the rest of the reference population. To achieve this result, we subsampled 50 subjects at random as test cases and then subsampled the other 150 subjects down to 100 training cases and repeated the procedure 30 times. In the 30 repeated procedures, using these randomly selected training sets, we applied the CLOUD conformity test with several values of *k* (number of nearest neighbors), from *k* = 1 to *k* = (all test cohort − 1) and did not identify any subjects as outliers, except for extreme values of *k* in several random datasets, demonstrating the robustness of the CLOUD test to neighborhood sizes and the low false-positive rate, as reported in Fig. 4a.

We also evaluated the CLOUD test on a previously published cohort of individuals from the Amazonas of Venezuela, rural Malawi and US metropolitan areas [28]. We only included the stool samples from the subjects older than 15 years old (*n* = 219). We used the unweighted UniFrac distance matrix of the 219 fecal samples. We subsampled 50 subjects at random as test cases and then subsampled the other 169 subjects down to 100 training cases and repeated the procedure 30 times. In the 30 repeated procedures, using random selected training datasets, we applied our dysbiosis test to several values of *k*, as described above, and found no outliers in any train/test subsets, as reported in Fig. 4b, c. This demonstrates the robustness of the CLOUD test to different training sets from a given reference population. Here, the test can successfully account for very high inter-individual variability as the subjects from different countries had highly divergent microbiomes.

### Application 2: microbiome restoration following FMT

In humans and murine models, fecal microbiota transplantation (FMT) has demonstrated high efficacy to cure CDI, a severe and relapsing infection with an increasing incidence rate [29, 30]. Several studies reported that the fecal microbiome of recipients following FMT was more diverse and more similar to the donor microbial community structure than the microbiome of the patient collected prior to transplantation [31, 32]. A recent study from our group showed that FMT resulted in rapid normalization of bacterial fecal sample composition from a markedly dysbiotic state to one representative of normal fecal microbiome in patients successfully treated with FMT [17]. However, there is no good statistical test to determine whether a patient’s microbiome has recovered relative to a population of healthy subjects. Here, we applied the CLOUD test to FMT recipients and compared them in terms of conformity to a group of healthy subjects.

#### Conformity and successful FMT

**Fig. 5:**
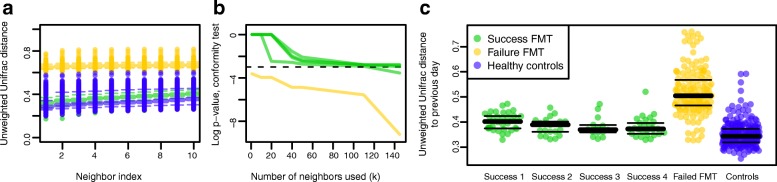
**a** The first 10 nearest independent healthy neighbors. This plot shows the restoration of the microbiome in responders to FMT, as the distances were very similar between samples from healthy subjects and those with successful FMT. This also shows failed restoration of the microbiome in the non-responder patient, as the distances were very different between the samples from failed patient and from the healthy subjects. **b** Plot of the log10 outlier percentile in patients who received FMT. The dashed line represents an outlier percentile of 0.05. When using *k* = 5% of the population, non-responder patient was considered as outlier. Using large neighborhood sizes classified 1 responder patient as outlier. **c** Patient stability as measured by self-similarity over time. Plot of the distance of a day to the corresponding previous day using Unweighted UniFrac distance. The figure shows stability between two consecutive samples of the fecal microbiome in healthy controls and in responder patients among days whereas the non-responder patient showed instability between two consecutive samples

We applied the CLOUD test to assess the successful microbiome restoration following FMT in our cohort of patients [17]. Results in Fig. 5a, which plots the 10 nearest independent healthy neighbors, show clear restoration to a non-dysbiotic microbiome in the patients who were cured of CDI following FMT, as the Unweighted UniFrac distances were very close in the two groups (healthy subjects and successful FMT) and the difference between the mean distance was not different. This also shows failed restoration of the microbiome by FMT in the patient who ultimately relapsed with CDI, as these distances were notably different between the two groups (healthy subjects and failed FMT, outlier percentile < 0.001). This shows the ability of CLOUD test to successfully differentiate between patients whose FMT ultimately resulted in success.

We then applied the CLOUD test on a per-patient level, that is, aggregating all samples from a single patient into a single averaged sample (responders and non-responders to FMT), and found that the four responder patients were not considered outliers, whereas the patient non-responsive to FMT was considered as an outlier. This conformity test is robust to neighborhood size as increasing the number of nearest independent healthy neighbors (from *k* = 1 to *k* = 100) always showed a significant difference between the healthy controls and the samples from the patient unsuccessfully treated with FMT, and no difference between the healthy controls and the samples from the patients who responded to the FMT, as showed in Fig. 5b. However, using very large *k* (*k* > 100), one responder patient was considered as an outlier (outlier percentile < 0.05). Again, this demonstrates the need for using only local neighborhoods of ecological distances, as reported in Fig. 3.

#### Conformity test in two other studies of FMT in recurrent CDI

We applied the CLOUD test to a published dataset describing recurrent CDI that explored the fecal microbiota of FMT stool donors and recipients [32]. This dataset includes 10 samples from donors, 14 pre-FMT samples from recipients, and 16 post-FMT samples. Specifically, 5 post-FMT samples tested positive for concomitant *Clostridium difficile* and 11 post-FMT samples tested negative. We used the cohort of donors to define the nearest independent healthy neighbors. Using Bray-Curtis distances, we tested all the samples collected from FMT recipients. We showed that with *k* corresponding to 5 to 40% of the number of healthy donor samples, the post-FMT samples which tested positive for concomitant *Clostridium difficile* were all considered outliers by CLOUD (outlier percentile < 0.001). Additionally, the pre-FMT samples were considered outliers (outlier percentile < 0.001) whereas the post-FMT samples testing negative for concomitant *Clostridium difficile* (outlier percentile = 0.4 to 0.75) were correctly classified as non-outliers.

We also applied the CLOUD test to another published fecal dataset that described the relationship between predictive signals from the gut microbiome and the development of recurrent CDI [33]. This dataset included 10 samples from donors, 11 recipient samples collected from patients who presented a recurrence of CDI, and 21 recipient samples from patients who did not present a recurrence as they were considered non-dysbiotic and cured. We used the cohort of donors from the same dataset to define the nearest independent healthy neighbors. Using the Bray-Curtis distances matrix of the fecal samples, we tested all the samples collected in the FMT recipients. As with the previous dataset, we showed that with a *k* corresponding to 5 to 40% of the number of healthy donor samples, the samples from the patients who presented a recurrence were all considered outliers by CLOUD (outlier percentile < 0.001), as were all pre-FMT recipient samples (outlier percentile < 0.001). In contrast, all samples from the patients who did not experience a recurrence were conformant (not considered outliers, outlier percentile = 0.6 to 0.65).

#### Stability test in the FMT data set

To assess the stability of the intestinal microbiome in patients cured of CDI by FMT, we obtained the CLOUD stability metric described above. We observed a high stability of the fecal microbiome in healthy controls over a daily time-course as well as in patients who successfully responded to FMT in the days following the FMT procedure. In contrast, the microbiome of the patient who did not respond to FMT showed dysbiosis and instability across the different sample time collections after the FMT procedure (Additional file 2). Results in Fig. 5c show high stability between consecutive daily samples of the fecal microbiome in healthy controls and in patients who responded successfully to FMT, whereas the patient who relapsed showed substantially higher instability on average between each two consecutive samples, although there were an insufficient number of independent reference subjects with multiple daily time points to obtain a reliable outlier percentile.

## Discussion

There is a large variability in terms of microbiome composition between individuals and within individuals over time [1, 2]. Methods have been developed to test whether disease conditions are associated or correlated with specific taxa or overall ecological community composition [3–7]. However, to our knowledge, there are no previously published non-parametric statistic tests of whether a patient’s overall microbiome profile can be considered healthy, based on a reference group of healthy individuals with widely varying microbiome profiles at a given moment or during a given period. Here, we developed a test for restoration from a dysbiotic state following FMT by characterizing the human gut microbiome that accounts for the wide range of microbiome phenotypes observed in a set of healthy individuals and for intra-individual temporal variation. This robust non-parametric test is based on local ecological distances and can be used to identify subjects with microbiomes that are significantly abnormal in terms of conformity or stability over time. Our test further allows unsupervised detection of microbiome outliers. We have validated the dysbiosis test in three clinical data sets to show concordance of the dysbiosis test with clinical outcomes of *Clostridium difficile* infection recurrence [17, 32, 33]. We have also demonstrated that local stability analysis within a subject’s microbiome cloud over time provides strong separation of patients who underwent successful and unsuccessful FMT procedures, with a failed procedure (defined as presence of later CDI relapse) resulting in a significantly less stable patient microbiome.

Such unsupervised identification of conformity and stability outliers in microbiome analyses is especially challenging for three reasons, which we have addressed in our method as follows. First, the human microbiome is highly multivariate, containing hundreds or thousands of different species within each individual. Our dysbiosis test uses ecologically and phylogenetically informed whole-microbiome distance metrics, such as UniFrac (for operational taxonomic units or OTUs) or Bray-Curtis (for species-level taxa) to assess the level of divergence of the mixtures of species or OTUs within two individuals, rather than focusing on any individual members of the microbiome.

Second, the healthy human gut microbiome has many different taxonomic configurations. Two humans can have almost completely different sets of bacteria in them and yet can still be considered healthy. Our method uses local measures of ecological distance only. We assess the ecological proximity of the test subject to that subject’s nearest healthy neighbors to determine the conformity of the test subject’s “personal microbiome cloud”. We then compare the cloud’s proximity to the clouds of all healthy individuals to determine whether the test subject is sufficiently close to at least some other healthy people to be considered healthy. Relying only on local ecological distances allows flexibility to account for the arbitrary topography and density distribution of the high-dimensional set of personal microbiome clouds of healthy individuals.

Third, an individual’s microbiome can vary substantially from day to day. We calculated the neighborhood sizes above not based on a single time point from each subject, but rather on the average across multiple time points to account for temporal variability in estimating a subject’s microbiome cloud conformity. Furthermore, we propose a separate test of the diameter of the test subject’s personal microbiome cloud and compared it to the distribution of diameters of the reference or healthy subjects’ microbiome clouds to evaluate stability.

Fourth, it is difficult to collect and store all samples in exactly the same way in a study, especially in longitudinal studies, where samples collected at the final time point may spend less time in frozen storage prior to DNA extraction than samples collected at other time points. In mouse studies, cage and animal batch effects can also introduce systematic biases. The CLOUD test may be a useful way to detect outliers in a study with problematic data linked to sample collection or preservation errors.

The CLOUD test has several limitations that are important to note. A key component of the approach is the choice of distance metric being used, as different distances posit different models of ecological similarity. Here, we used Unweighted UniFrac distance metric as we were analyzing 16S data and UniFrac distance are an effective distance metric in this case. However, other ecological distances may be appropriate for certain studies, and indeed, we found that Bray-Curtis worked well with the CLOUD test in discriminating between recovery and non-recovery in two recurrent CDI data sets. The CLOUD test also requires that the reference set be chosen properly to represent sufficient variation in the high-dimensional reference microbiome landscape, and that the test samples be collected and analyzed in the same way as the reference samples.

## Conclusions

As the medical microbiome research field moves closer to translation from epidemiological surveys to clinical applications, clinicians need a robust measure that can determine whether a microbiome is statistically similar to microbiomes in a reference population. This measure must account for the high dimensionality, high inter-individual variability, and high longitudinal variability of the microbiome. The CLOUD test is designed to account for these constraints and is useful for comparing a patient’s microbiome to a reference population to determine whether it is significantly abnormal or dysbiotic in terms of conformity or stability [34]. The test is reliant on having a relevant reference cohort of healthy individuals but is also entirely invariant to the addition or removal of highly discordant samples from the database due to its reliance on local distances. The ability to detect conformity- or stability-related dysbiosis may become useful as a diagnostic tool in a variety of medical conditions associated with altered functionality of microbiome in pediatric or adult clinical practice.

## Additional files

Additional file 1: R code for calculating the neighborhood diameter. (DOCX 58 kb)

Additional file 2: Patient stability as measured by self-similarity over time. Plot of the distance of a day using Unweighted UniFrac distance of the 4 patients who succeed FMT, one patient who failed FMT and 16 healthy controls. Samples were collected from day 1 to day 150. The plot does not include the preFMT samples in FMT-recipient patients. The figure shows stability between samples of the fecal microbiome in healthy controls and in patients with successful FMT among days whereas the patient who failed FMT showed instability between two consecutive samples. (PDF 193 kb)

## Abbreviations

CDI: *Clostridium difficile* infection
FMT: Fecal microbiota transplantation
IBD: Inflammatory bowel disease
OTU: Operational taxonomic unit
PC: Principal component
PCoA: Principal coordinates analysis
QIIME: Quantitative Insights Into Microbial Ecology

## References

[1] Zhernakova A, Kurilshikov A, Bonder MJ, Tigchelaar EF, Schirmer M, Vatanen T (2016). Population-based metagenomics analysis reveals markers for gut microbiome composition and diversity. Science.

[2] Falony G, Joossens M, Vieira-Silva S, Wang J, Darzi Y, Faust K (2016). Population-level analysis of gut microbiome variation. Science.

[3] Segata N, Izard J, Waldron L, Gevers D, Miropolsky L, Garrett WS, Huttenhower C (2011). Metagenomic biomarker discovery and explanation. Genome Biol..

[4] Kuczynski J, Stombaugh J, Walters WA, González A, Caporaso JG, Knight R. Using QIIME to analyze 16S rRNA gene sequences from microbial communities. Curr. Protoc. Bioinforma. 2011; 10.1002/0471250953.bi1007s36. Chapter 10:Unit 10.7. Ed. Board Andreas Baxevanis Al 10.1002/9780471729259.mc01e05s27 PMC4477843 23184592

[5] Paulson JN, Stine OC, Bravo HC, Pop M (2013). Differential abundance analysis for microbial marker-gene surveys. Nat Methods.

[6] McMurdie PJ, Holmes S (2014). Waste not, want not: why rarefying microbiome data is inadmissible. PLoS Comput Biol.

[7] McMurdie PJ, Holmes S (2015). Shiny-phyloseq: Web application for interactive microbiome analysis with provenance tracking. Bioinforma Oxf Engl.

[8] Halfvarson J, Brislawn CJ, Lamendella R, Vázquez-Baeza Y, Walters WA, Bramer LM (2017). Dynamics of the human gut microbiome in inflammatory bowel disease. Nat Microbiol.

[9] Vandeputte D, Falony G, Vieira-Silva S, Tito RY, Joossens M, Raes J (2016). Stool consistency is strongly associated with gut microbiota richness and composition, enterotypes and bacterial growth rates. Gut.

[10] Human Microbiome Project Consortium (2012). Structure, function and diversity of the healthy human microbiome. Nature.

[11] Arumugam M, Raes J, Pelletier E, Le Paslier D, Yamada T, Mende DR (2011). Enterotypes of the human gut microbiome. Nature.

[12] Wang J, Linnenbrink M, Künzel S, Fernandes R, Nadeau M-J, Rosenstiel P (2014). Dietary history contributes to enterotype-like clustering and functional metagenomic content in the intestinal microbiome of wild mice. Proc Natl Acad Sci U S A.

[13] Knights D, Ward TL, McKinlay CE, Miller H, Gonzalez A, McDonald D, et al. Rethinking “enterotypes.” Cell Host Microbe 2014;16:433–437. 10.1016/j.chom.2014.09.013 PMC5558460 25299329

[14] Zamanzad Ghavidel F, Claesen J, Burzykowski T, Valkenborg D (2014). Comparison of the Mahalanobis distance and Pearson’s χ^2^ statistic as measures of similarity of isotope patterns. J Am Soc Mass Spectrom.

[15] Suzuki H, Sota M, Brown CJ, Top EM (2008). Using Mahalanobis distance to compare genomic signatures between bacterial plasmids and chromosomes. Nucleic Acids Res.

[16] Todeschini R, Ballabio D, Consonni V, Sahigara F, Filzmoser P (2013). Locally centred Mahalanobis distance: a new distance measure with salient features towards outlier detection. Anal Chim Acta.

[17] Weingarden A, González A, Vázquez-Baeza Y, Weiss S, Humphry G, Berg-Lyons D (2015). Dynamic changes in short- and long-term bacterial composition following fecal microbiota transplantation for recurrent Clostridium difficile infection. Microbiome.

[18] Hamilton MJ, Weingarden AR, Sadowsky MJ, Khoruts A (2012). Standardized frozen preparation for transplantation of fecal microbiota for recurrent Clostridium difficile infection. Am J Gastroenterol.

[19] Caporaso JG, Kuczynski J, Stombaugh J, Bittinger K, Bushman FD, Costello EK (2010). QIIME allows analysis of high-throughput community sequencing data. Nat Methods.

[20] Caporaso JG, Lauber CL, Walters WA, Berg-Lyons D, Huntley J, Fierer N (2012). Ultra-high-throughput microbial community analysis on the Illumina HiSeq and MiSeq platforms. ISME J..

[21] Bokulich NA, Subramanian S, Faith JJ, Gevers D, Gordon JI, Knight R (2013). Quality-filtering vastly improves diversity estimates from Illumina amplicon sequencing. Nat Methods.

[22] McDonald D, Price MN, Goodrich J, Nawrocki EP, DeSantis TZ, Probst A (2012). An improved Greengenes taxonomy with explicit ranks for ecological and evolutionary analyses of bacteria and archaea. ISME J.

[23] Lozupone C, Knight R (2005). UniFrac: a new phylogenetic method for comparing microbial communities. Appl Environ Microbiol.

[24] R Core Team (2014). R: a language and environment for statistical computing. R Foundation for Statistical Computing, Vienna. URL http://www.R-project.org/.

[25] Grubbs FE (1969). Procedures for detecting outlying observations. Technometrics.

[26] Tietjen GL, Moore RH (1972). Some Grubbs-type statistics for the detection of outliers. Technometrics.

[27] Rosner B (1983). Percentage points for a generalized ESD many-outlier procedure. Technometrics.

[28] Yatsunenko T, Rey FE, Manary MJ, Trehan I, Dominguez-Bello MG, Contreras M (2012). Human gut microbiome viewed across age and geography. Nature.

[29] Gough E, Shaikh H, Manges AR (2011). Systematic review of intestinal microbiota transplantation (fecal bacteriotherapy) for recurrent Clostridium difficile infection. Clin Infect Dis Off Publ Infect Dis Soc Am.

[30] van Nood E, Vrieze A, Nieuwdorp M, Fuentes S, Zoetendal EG, de Vos WM (2013). Duodenal infusion of donor feces for recurrent Clostridium difficile. N Engl J Med.

[31] Seekatz AM, Theriot CM, Molloy CT, Wozniak KL, Bergin IL, Young VB (2015). Fecal microbiota transplantation eliminates Clostridium difficile in a murine model of relapsing disease. Infect Immun.

[32] Seekatz AM, Aas J, Gessert CE, Rubin TA, Saman DM, Bakken JS (2014). Recovery of the gut microbiome following fecal microbiota transplantation. MBio.

[33] Seekatz AM, Rao K, Santhosh K, Young VB (2016). Dynamics of the fecal microbiome in patients with recurrent and nonrecurrent Clostridium difficile infection. Genome Med.

[34] Wang F, Kaplan JL, Gold BD, Bhasin MK, Ward NL, Kellermayer R (2016). Detecting microbial dysbiosis associated with pediatric Crohn’s disease despite the high variability of the gut microbiota. Cell Rep.

