# Supplementary material for: CLOUD: a non-parametric detection test for microbiome outliers
Source: Microbiome. 2018 Aug 6;6:137. doi: 10.1186/s40168-018-0514-4 (PMC6080375; doi:10.1186/s40168-018-0514-4)
Supplement: Supplementary file 1 — R code for calculating the neighborhood diameter. (DOCX 58 kb) [file 40168_2018_514_MOESM1_ESM.docx]

**Additional file 1. R code for calculating the neighborhood diameter**

# inputs a distance matrix

# returns piecewise distances of samples and their outlier percentile

# and a matrix of the repeated measures of distances

# k is number of neighbors choosen

"piecewise_kn_V1" <- function(d, test.ix, k=X, ndim=-1){

if(class(d) != 'matrix') d <- as.matrix(d)

stats <- numeric(length(test.ix))

pvals <- numeric(length(test.ix))

for(i in 1:length(test.ix)){

ref.ix <- test.ix[-i]

keep.ix <- c(test.ix[i], ref.ix)

if(ndim > -1){

pc <- cmdscale(d[keep.ix,keep.ix,drop=F],k=ndim)

d.i <- as.matrix(dist(pc))

} else {

d.i <- d[keep.ix,keep.ix,drop=F]

}

test.dist <- mean(sort(d.i[1,-1])[1:k])

ref.dists <- numeric(length(ref.ix))

for(j in 1:length(ref.ix)){

ref.dists[j] <- mean(sort(d.i[-1,-1][j,-j]))

}

stats[i] <- test.dist / mean(ref.dists)

pvals[i] <- mean(test.dist < ref.dists)

}

result <- list()

result$stats <- stats

result$pvals <- pvals

outcome <- pvals <= 0.05

result$lenght <- length(outcome[outcome==TRUE])

return(result)

}
