# Supplementary material for: CLOUD: a non-parametric detection test for microbiome outliers
Source: Microbiome. 2018 Aug 6;6:137. doi: 10.1186/s40168-018-0514-4 (PMC6080375; doi:10.1186/s40168-018-0514-4)
Supplement: Supplementary file 2 — Patient stability as measured by self-similarity over time. Plot of the distance of a day using Unweighted UniFrac distance of the 4 patients who succeed FMT, one patient who failed FMT and 16 healthy controls. Samples were collected from day 1 to day 150. The plot does not include the preFMT samples in FMT-recipient patients. The figure shows stability between samples of the fecal microbiome in healthy controls and in patients with successful FMT among days whereas the patient who failed FMT showed instability between two consecutive samples. (PDF 193 kb) [file 40168_2018_514_MOESM2_ESM.pdf]

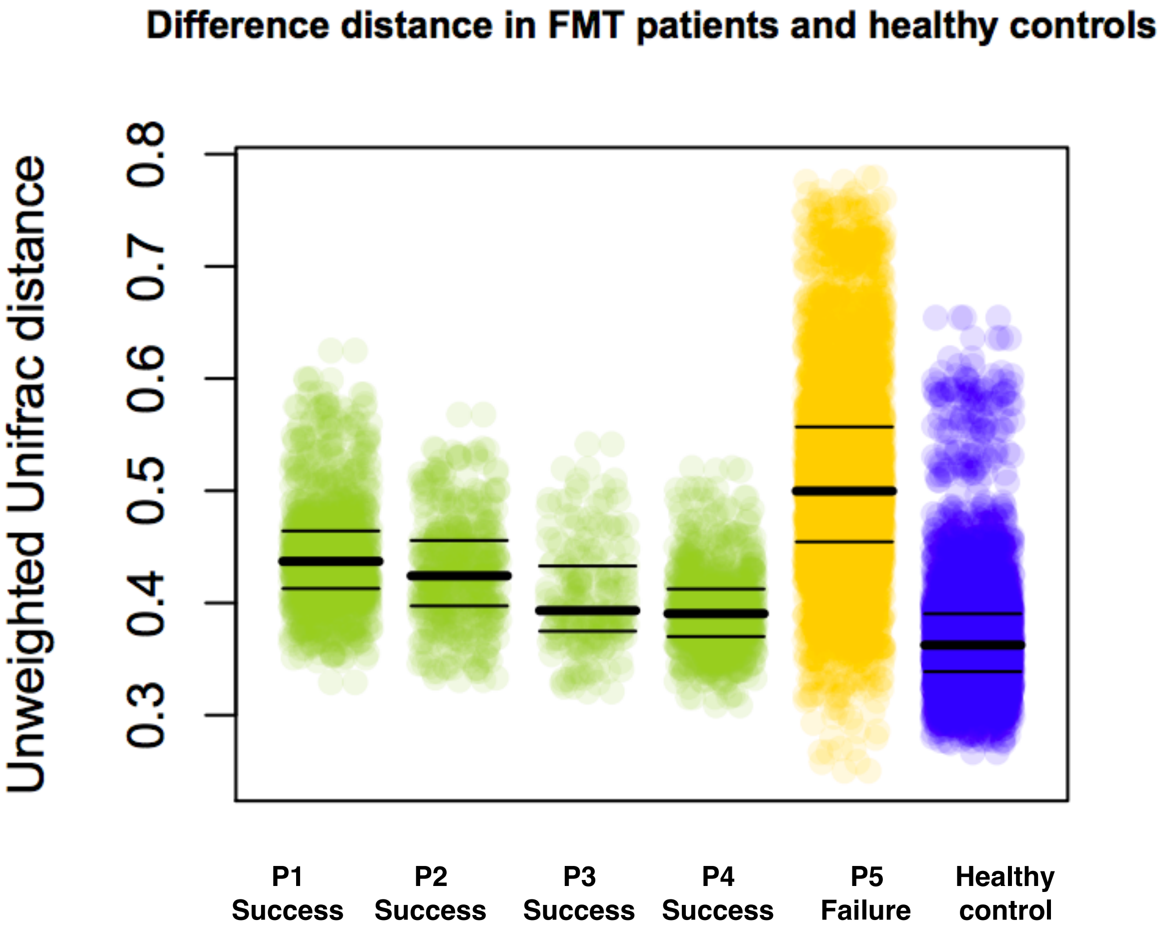

Additional file 3. Patient stability as measured by self-similarity over time. Plot of the distance of a day using Unweighted UniFrac distance of the 4 patients who succeed FMT, one patient who failed FMT and 16 healthy controls. Samples were collected from Day 1 to Day 150. The plot does not include the preFMT samples in FMT recipients patients. The figure shows stability between samples of the fecal microbiome in healthy controls and in patients with successful FMT among days whereas the patient who failed FMT showed instability between two consecutive samples.
